# Quantitative proteomics of a B_12_‐dependent alga grown in coculture with bacteria reveals metabolic tradeoffs required for mutualism

**DOI:** 10.1111/nph.14832

**Published:** 2017-10-16

**Authors:** Katherine E. Helliwell, Jagroop Pandhal, Matthew B. Cooper, Joseph Longworth, Ulrich Johan Kudahl, David A. Russo, Eleanor V. Tomsett, Freddy Bunbury, Deborah L. Salmon, Nicholas Smirnoff, Phillip C. Wright, Alison G. Smith

**Affiliations:** ^1^ Department of Plant Sciences University of Cambridge Cambridge CB2 3EA UK; ^2^ Department of Chemical and Biological Engineering University of Sheffield Mappin Street Sheffield S1 3JD UK; ^3^ Biosciences College of Life and Environmental Sciences University of Exeter Exeter EX4 4QD UK

**Keywords:** iTRAQ proteomics, *Lobomonas rostrata*, *Mesorhizobium loti*, mutualism, photosynthesis, vitamin B_12_

## Abstract

- The unicellular green alga *Lobomonas rostrata* requires an external supply of vitamin B_12_ (cobalamin) for growth, which it can obtain in stable laboratory cultures from the soil bacterium *Mesorhizobium loti* in exchange for photosynthate. We investigated changes in protein expression in the alga that allow it to engage in this mutualism.
- We used quantitative isobaric tagging (iTRAQ) proteomics to determine the *L. rostrata* proteome grown axenically with B_12_ supplementation or in coculture with *M*. *loti*. Data are available via ProteomeXchange (PXD005046).
- Using the related *Chlamydomonas reinhardtii* as a reference genome, 588 algal proteins could be identified. Enzymes of amino acid biosynthesis were higher in coculture than in axenic culture, and this was reflected in increased amounts of total cellular protein and several free amino acids. A number of heat shock proteins were also elevated. Conversely, photosynthetic proteins and those of chloroplast protein synthesis were significantly lower in *L. rostrata* cells in coculture. These observations were confirmed by measurement of electron transfer rates in cells grown under the two conditions.
- The results indicate that, despite the stability of the mutualism, *L. rostrata* experiences stress in coculture with *M. loti*, and must adjust its metabolism accordingly.

## Introduction

Microalgae are a polyphyletic set of photosynthetic eukaryotes found across the eukaryotic tree of life which are estimated to be responsible for *c*. 50% of global CO_2_ fixation (Falkowski, 1998). In both soil and aquatic (marine and freshwater) habitats, algae exist alongside a wide spectrum of other microbes, including bacteria, archaea, fungi and cyanobacteria. This has led to millions of years of coevolution between contemporaneous species. It is therefore not surprising that a broad range of interactions between different microbial players within the ecosystem have been observed. As well as harmful interactions (Fernandes *et al*., 2011), many examples of beneficial associations have been documented between algae and bacteria, including promotion of algal growth (Park *et al*., 2008), support of cellular differentiation (Matsuo *et al*., 2005), provision of bactericidal/algaecidal protection (Hold *et al*., 2001; Geng *et al*., 2008; Thiel *et al*., 2010), stress tolerance (Xie *et al*., 2013), and hormonal stimulation (Ashen *et al*., 1999; Amin *et al*., 2015). Underpinning many mutualistic interactions is nutrient exchange (Cooper & Smith, 2015), where algal photosynthate is exchanged for micronutrients provided by bacteria, for example by facilitation of iron uptake (Amin *et al*., 2009), or provision of the vitamins thiamine (vitamin B_1_) (Paerl *et al*., 2015) or cobalamin (vitamin B_12_) (Croft *et al*., 2005; Wagner‐Döbler *et al*., 2010; Kazamia *et al*., 2012; Durham *et al*., 2015). Vitamins are organic micronutrients, which perform essential functions within the cell as enzyme cofactors. Increasingly, it is recognized that many algae, despite their photosynthetic lifestyles, require an exogenous source of one or more of these compounds, or their precursors, for growth (reviewed in Helliwell, 2017). Analysis of environmental samples has detected the co‐occurrence of bacterial producers and algal requirers of cobalamin in a variety of marine environments (Koch *et al*., 2013; Bertrand *et al*., 2015), and fertilization experiments have demonstrated that these compounds are limiting for algal productivity (Koch *et al*., 2012), providing evidence that algal–bacterial interactions are likely to be widespread and of considerable significance for global net primary production.

Cobalamin is required by over 50% of all microalgal species surveyed (Croft *et al*., 2005), but with no phylogenetic relationship between dependent and nondependent species, implying that this trait has arisen multiple times during algal evolution. The requirement for cobalamin is as a cofactor for B_12_‐dependent methionine synthase (METH), which is involved in the synthesis of the amino acid methionine, as well as more generally in cellular C1 metabolism. Species that do not require vitamin B_12_ have an alternative, B_12_‐independent, enzyme, METE (Helliwell *et al*., 2011), which performs the same function, albeit at lower efficiency (González *et al*., 1992; Croft *et al*., 2005; Helliwell *et al*., 2015). Several species of algae, such as *Chlamydomonas reinhardtii* and *Phaeodactylum tricornutum*, encode both isoforms of methionine synthase, and can use METE when B_12_ is not available. The presence of an external supply of the vitamin allows these species to use METH, and at the same time *METE* gene expression is repressed, in both laboratory cultures (Helliwell *et al*., 2011; Bertrand *et al*., 2012) and environmental samples (Bertrand *et al*., 2015). Long‐term exposure to B_12_ in the environment might therefore lead to loss of the *METE* gene, and indeed an *metE* mutant was generated by an experimental evolution approach after growth of *C. reinhardtii* cells in B_12_ for *c*. 500 generations (Helliwell *et al*., 2015). It is conceivable, therefore, that interactions with B_12_‐producing bacteria in the environment over evolutionary time might lead to the frequent loss of METE in diverse algal lineages (Kazamia *et al*., 2016), which would account for the widespread occurrence of B_12_ auxotrophy across the algal lineages.

A major challenge now is to dissect the specific molecular mechanisms that underpin the exchange of cobalamin, but this is difficult in the dilute conditions of the aquatic environment. To study aspects such as signalling, regulation, transporter proteins and dynamics over time, defined model systems are required. In our laboratory we developed a model partnership between the vitamin B_12_‐requiring freshwater green alga *Lobomonas rostrata*, a close relative of *C. reinhardtii*, and the rhizobial bacterium *Mesorhizobium loti*, which supplies vitamin B_12_ in exchange for fixed carbon (Kazamia *et al*., 2012). Although a synthetic interaction, the ease with which it formed mirrors that of other artificial systems, such as the mutualism established between *C. reinhardtii and* the yeast *Saccharomyces cerevisiae* based on the exchange of carbon and nitrogen (Hom & Murray, 2014). Moreover, physiological experiments with our *L. rostrata* and *M. loti* cocultures under both batch and semicontinuous conditions revealed that an equilibrium in terms of cell number was established and maintained (Kazamia *et al*., 2012), demonstrating that the partnership exhibits a degree of regulation. The direct exchange of B_12_ could be modelled using growth dynamics of algal–bacterial cocultures (Grant *et al*., 2014), and the model implicated specific release from live bacterial cells, rather than simply release of the vitamin once the cells had died and lysed. With this observation of true mutualism between the algae and bacteria, we might expect specific metabolic changes in *L. rostrata* when grown in coculture, as compared with axenic growth with B_12_ supplementation.

Genome‐scale approaches to dissect metabolic shifts occurring on account of interspecies microbial interactions have been applied to a variety of different phytoplankton–bacteria coculture systems. Such work has revealed important insights into alterations in core metabolism, including amino acid biosynthesis in associations between cyanobacteria and heterotrophic bacteria (Beliaev *et al*., 2014; Biller *et al*., 2016), as well as sulphur cycling (Durham *et al*., 2015), and signalling and production of infochemicals (Amin *et al*., 2015) in diatom–bacterial interactions. However, the majority of the studies were at the transcript level, whereas it is proteins that are responsible for performing cellular processes. A requirement for proteomics analyses is the need for databases of peptides that can be matched to experimental mass spectra. Until recently, this required annotated genome sequence information of the organism under study, which is not currently available for *L. rostrata*, but as more organisms have been sequenced, this has resulted in many more shared peptides in the databases. In this study, we performed a series of preliminary bioinformatics and proteomics experiments to evaluate the applicability of the global quantitative proteomics chemical isobaric tags (iTRAQ) methodology to our system. Once the feasibility was confirmed, the approach was used to identify metabolic differences between *L. rostrata* cells grown in media supplemented with vitamin B_12_ and those grown in cultures where B_12_ is provided by *M. loti* in a mutualistic exchange. This has provided novel insights into the metabolic consequences of mutualism between a photosynthetic green alga and its heterotrophic bacterial partner.

## Materials and Methods

### Algal and bacterial strains and cultivation

Lobomonas *rostrata* (SAG 45‐1) was obtained from the Experimental Phycology and Culture Collection of Algae at the University of Goettingen (EPSAG), Germany. It was grown autotrophically on TP^+^ medium (Kazamia *et al*., 2012). Vitamin B_12_ was provided as cyanocobalamin (Sigma‐Aldrich) at 100 ng l^−1^, as this supports the maximum carrying capacity of *L. rostrata* (Kazamia *et al*., 2012). The *L. rostrata–M. loti* coculture was an established coculture that had been maintained over many generations without a source of organic carbon or vitamin B_12_. Cultures were maintained on a 16 : 8 h, light : dark cycle, with shaking (140 rpm) at 25°C. *M. loti* (MAFF 303099) was a gift from Prof. Allan Downie at the John Innes Centre, Norwich, UK. It was maintained axenically in TP+ with 0.1% v/v glycerol at 28°C. Cells were harvested by centrifugation, and, if not analysed immediately, cell pellets were frozen in liquid N_2_ and stored at −80°C. Algal cell counts were determined using a Dual Threshold Beckman Coulter (Z2) Particle Counter and Size Analyser (Indianapolis, IN, USA). Bacterial cell numbers were determined by plating on solid media.

### Protein preparation and iTRAQ labelling

Full experimental details are provided in Supporting Information Methods S1. In brief, cultures were harvested at 3000 ***g*** for 10 min at 4°C. The resulting cell pellets were washed in 0.5 M triethylammonium bicarbonate buffer, and then extracted by grinding in liquid nitrogen and sonication. Insoluble protein was removed by centrifugation. The soluble protein fraction was precipitated using acetone, then the resuspended pellet was reduced with tris‐(2‐carboxyethyl)‐phosphine, followed by alkylation with methyl methanethiosulfonate in isopropanol. Samples were digested with trypsin and the resulting peptides were either taken forward for mass spectrometry analysis for preliminary proteomics analysis or labelled with iTRAQ reagents following the manufacturer's instructions (AB Sciex, Famingham, MA, USA). Labels 113, 114, 115 and 116 were used to label biological replicates of *L. rostrata* cells cultured with B_12_ supplemented in the media, and labels 117, 118, 119 and 121 were used for *L. rostrata* cells cultured with *M. loti*.

### Hydrophilic interaction liquid chromatography and mass spectrometry

All eight labelled peptide samples were combined before being dried in a vacuum concentrator (Concentrator 5301; Eppendorf, Stevenage, UK). The sample was resuspended in 200 μl of buffer A (10 mM ammonium formate, 90% ACN, pH 3 (adjusted with formic acid)) and 100 μl was loaded onto a PolyHydroxyethyl A column (particle size, 5 μm; length, 20 cm; diameter, 2.1 mm; pore size, 200 Å; PolyLC, Colombia, MD, USA) using an Agilent 1100‐series HPLC (Agilent, Wokingham, UK). With a flow of 0.5 ml min^−1^, buffer A was exchanged with buffer B (10 mM ammonium formate, 10% ACN, pH 4 (adjusted with formic acid)) to form a linear gradient as follows: 0% B (0–5 min), 0–15% B (5–7 min), 15% B (7–10 min), 15–60% B (10–50 min), 60–100% B (50–55 min), 100% B (55–65 min), 0% B (65–75 min). Beginning at 18 min, 22 fractions of 1 min length, followed by three fractions of 3 min length were collected and dried by vacuum centrifugation ready for reverse‐phase LC‐MS/MS.

Liquid chromatography‐tandem mass spectrometry was conducted using an Ultimate 3000 high‐performance liquid chromatograph (Dionex, Sunnyvale, CA, USA) coupled to a QStar XL Hybrid electrospray ionization (ESI) quadrupole time‐of‐flight tandem mass spectrometer (Applied Biosystems (now ABSciex), Framingham, MA, USA). Samples were resuspended in 20 μl buffer A (3% ACN, 0.1% FA) before loading 9 μl onto a Acclaim PepMap 100 C18 column (particle size, 3 μm; length, 15 cm; diameter, 75 μm; pore size, 100 Å; Dionex, Sunnyvale, CA, USA). With a flow of 300 μl min^−1^ buffer A was exchanged with buffer B (97% ACN, 0.1% FA) to form a linear gradient as follows. 3% B (05 min), 3–35% B (5–95 min), 35–90% B (95–97 min), 90% B (97–102 min), 3% B (102–130 min). The mass detector range was set to 350–1800 *m/z* and operated in positive ion mode. Peptides with +2, +3, and +4 were selected for fragmentation. The mass spectrometry proteomics data have been deposited to the ProteomeXchange Consortium via the PRIDE (Vizcaíno *et al*., 2014) partner repository with the dataset identifier PXD005046.

### Protein identification and quantification

The work flow for proteomic identifications is shown in Fig. S1. Searches were conducted using Mascot, Ommsa, X!Tandem, Peaks and ProteinPilot against the Uniprot reference proteome for *C. reinhardtii* (Uniprot ID 3055) and *M. loti* (Uniprot ID 266835). Each search was conducted with the target‐decoy database method to calculate false discovery rate (FDR; Elias & Gygi, 2007). The decoy was formed using either reversed sequences (Mascot, Ommsa, X!Tandem and ProteinPilot) or randomized sequence (Peaks) of the *C. reinhardtii* and *M. loti* proteomes. Searches were restricted to a peptide FDR of 3% before decoy hits were removed at the peptide spectral match (PSM) level. PSMs are the individual matches made by the search engine algorithm between the mass spectrometer's output product ion scan and the potential peptides contained within the search database. They combine to form the peptide and protein identifications. PSMs from the five search engines were merged using an R‐based script that also removed PSMs showing disagreement in terms of peptide assignment or protein identification between the search engines, as performed previously (Longworth *et al*., 2016). Separately, reporter ion intensities for each PSM were extracted and matched to the merged results. For quantitation, these reporter ion intensities were extracted followed by variance stabilization normalization, isotopic correction and median correction, and finally averaging by protein. To avoid using arbitrary thresholds for highlighting differentially expressed proteins, a *t*‐test was performed between replicate conditions to determine significance with fold change. Transmembrane domains were identified using Tmhmm server 2.0 (http://www.cbs.dtu.dk/services/TMHMM/). We identified all putative transmembrane domains in each of the proteins identified in the iTRAQ experiment. If a sequence had one or more transmembrane domains, this was classified as likely to be membrane‐associated rather than soluble.

### Analytical methods

Maximum potential photosynthetic capacity as electron transfer rate (ETR) was estimated using pulse amplitude modulation (PAM). Cells were prepared to a density of 2.5 × 10^6^ cells ml^−1^, placed in cuvettes and mixed with a magnetic stirrer in order to maintain an even cell suspension. Instant light response curves of Chl fluorescence were generated for each sample using a Mini‐PAM (Heinz Walz GmbH, Effeltrich, Germany). The ETR was calculated as follows:

(Eqn 1) ETR=Y/1000×PAR×0.5×ETR factor,

where *Y*/1000 is equal to (*F*_m_′ − *F*)/*F*_m_′, or Φ_PSII_, the quantum yield of photosystem II. The ETR factor described refers to the fraction of incident photons absorbed by the sample, for which a default value of 0.83 was used (Maxwell & Johnson, 2000).

Protein was measured using Bradford's reagent as previously described (Bradford, 1976) using BSA as a standard. For amino acid analysis, full experimental details are provided in the Supporting Information Methods S1. In brief, *c*. 5 mg of a freeze‐dried sample of cultures were extracted in acetonitrile, and then re‐extracted with 20% methanol spiked with an internal standard containing stable isotope‐labelled amino acids (l‐amino acid mix; Sigma‐Aldrich). The supernatants were pooled and amino acids were derivatised with an AccQ•Tag Ultra derivatization kit (Waters Corp., Milford, MA, USA). HPLC‐ESI‐MS/MS quantitative analysis of the amino acids was performed using an Agilent 6420B triple quadrupole (QQQ) mass spectrometer (Agilent Technologies, Palo Alto, CA, USA). All ions were scanned in positive ion mode and given a dwell time of 50 ms. Data analysis was undertaken using Agilent Mass Hunter Quantitative analysis software for QQQ (v.B.07.01). Accurate quantification used the stable isotope‐labelled internal standards added during sample extraction, and data were normalized to the DW of the samples.

### Reverse transcriptase‐quantitative polymerase chain reaction (RT‐qPCR)

RNA was extracted from the cell pellet from 10 ml of day 14 (late‐log) cultures using RNeasy Plant Mini Kit (Qiagen), treated with Turbo DNA‐free™ kit (Ambion) to remove genomic DNA, and reverse‐transcribed with the Superscript III First‐trand synthesis system for RT‐PCR (Invitrogen). Selected *C. reinhardtii* coding sequences from the NCBI database were aligned with scaffolds produced from *L. rostrata* RNA‐seq data (U.J. Kudahl *et al*., unpublished) in order to find genes of interest; homologues were found to be 90% similar at the nucleotide level. Primers were designed with melting temperatures of 61–63°C, and to amplify products of 150–200 nucleotides (Untergasser *et al*., 2012), and the sequences are shown in Table S1. Quantitative PCR reactions were prepared in 10 μl volumes using SYBR Green JumpStart™ Taq ReadyMix™ (Sigma‐Aldrich). Reactions were performed in a Rotor‐Gene Q (Qiagen) with an initial denaturation at 95°C for 2 min followed by cycling 45 times between 58°C for 30 s and 95°C for 15 s, and finally a melt curve was generated by increasing the temperature from 55 to 95°C in 1°C steps held for 5 s. Amplification efficiency and cycle threshold values were calculated with the Rotor‐Gene Q software v.2.02. Quantification and normalization were carried out using the Pfaffl model (Pfaffl, 2001) with three reference genes: Ubiquitin (UBQ), Eukaryotic translation initiation factor 4A (EIF4A) and Receptor of activated protein kinase C1 (RACK1) (Mus *et al*., 2007).

## Results

### Preliminary bioinformatics and proteomic analyses

To ascertain whether it was feasible to use iTRAQ for global analysis of the proteome of unsequenced *L. rostrata*, a series of preliminary experiments was carried out. The first aim was to find a suitable reference proteome for matching experimental spectra sourced from *L. rostrata*. This process relies on shared peptides from sequenced organisms being present within existing protein databases. The closely related freshwater green alga *C. reinhardtii* (Uniprot ID 3055) was a prime candidate and also has the advantage of having a relatively well annotated genome (https://phytozome.jgi.doe.gov/pz/portal.html). A search against *C. reinhardtii* produced 1% PSMs, similar to a previous cross‐species proteomics study (Pandhal *et al*., 2008), implying that sufficient proteins could be identified in a global proteomics study to uncover biological insights into the metabolic processes of the unsequenced alga.

Our iTRAQ experiment was aimed at identifying proteins from organisms in coculture, so proteins from both *L. rostrata* and *M. loti* would be present. Therefore, it was important to consider the possibility that shared peptides between the two organisms might influence the results, namely that peptides from *M. loti* might be assumed to be from *L. rostrata*, and thus interfere with protein quantifications. To evaluate this, a theoretical tryptic digest was undertaken. The identification of shared peptides between the alga and bacteria was done using protein sequences for *M. loti* and *C. reinhardtii* retrieved from Uniprot (*02‐07‐2016* ). A python script was written to read the protein sequences and theoretically digest them into tryptic peptides, by cleaving them after arginine and lysine unless followed by a proline. Peptides between six and 16 amino acids long were identified and compared, calculating the shared fraction using the total number of unique peptides in *C. reinhardtii*. The result was that 0.31% of tryptic peptides within this size range were shared between *C. reinhardtii* and *M. loti*. Given this low number, and the close phylogenetic relationship between *C. reinhardtii* and *L. rostrata*, it was unlikely that interpretation of the iTRAQ experiment would be confounded by peptides shared between the alga and bacterium in the cocultures.

Finally, the iTRAQ approach rests on the relative quantification of peptides across samples, with the general assumption that proteins are present in all samples, whereas in our study comparing cocultures and axenic cultures of *L. rostrata*, only half would contain *M. loti* cells. Moreover, the aim of the experiment was to look for metabolic differences in *L. rostrata* under the two conditions, and therefore it would be ideal to minimize spectra sourced from *M. loti*, which could potentially reduce the total alga spectra generated per sample injection. This was potentially problematic, as the ratio of cell counts for *L. rostrata* to *M. loti* cells in stable cocultures is between 1 : 30 and 1 : 100 (Kazamia *et al*., 2012), averaging 1 : 50 in the samples used for the iTRAQ experiment here. Moreover, proteins from sequenced organisms are more likely to be identified compared with those from unsequenced organisms, in bottom‐up proteomics experiments. The latter was confirmed with proteins from axenic cultures of *M. loti*, where the PSM was 11% (compared with 1% PSM for *L. rostrata*). However, the much larger algal cell volume (*c*. 500 μm^3^, compared with *c*. 0.5 μm^3^ for the bacteria) means that in the coculture this would correspond to significantly more algal protein than bacterial protein, even with the higher number of bacterial cells. In order to test this, a protein per cell measurement was made for axenic cultures of the two organisms*,* and found to be 2.29 × 10^−7^ **mg cell^−1^ for *L. rostrata* and 3.25 × 10^−10 ^mg cell^−1^ for *M. loti*. At the cell densities in the coculture, this corresponds to *c*. 0.45 mg ml^−1^ for *L. rostrata* protein, compared with 0.033 mg ml^−1^ for *M. loti*, a 14‐fold higher amount. This, together with the nature of peptide fractionation in bottom‐up mass spectrometry‐based proteomics, where there is a bias towards identification of high‐abundance peptides and thus proteins, provided confidence in our approach.

### Overview of the *L. rostrata* proteome determined by iTRAQ analysis

Having established the parameters and methodology for the experiment, four biological replicates of both axenic *L. rostrata* supplemented with 100 ng l^−1^ B_12_ and established cocultures of *L. rostrata* and *M. loti* were grown in autotrophic medium. Cells were harvested after 14 d when the algae were at mid‐to‐late exponential growth phase (Fig. S2), and total soluble protein was extracted for proteomic analysis. Altogether, 588 proteins were identified based on one high‐confidence peptide hit, and about half of these (293) had a minimum of two high‐confidence peptide hits per protein. A total of 47 proteins were predicted to be membrane‐associated by virtue of the presence of at least one transmembrane domain. A search of the same mass spectral data against the *M. loti* database (Uniprot ID 266835) identified just four proteins (all based on one high‐confidence peptide hit), indicating that the vast majority of proteins identified were of algal origin. Comparison of proteins from the two different conditions revealed 153 proteins that were significantly differentially expressed (*P *>* *0.05). Of these, 70 were present in higher amounts in *L. rostrata* when grown with *M. loti* in comparison to the axenic condition, and 83 were less abundant. A heat map representation of these data showing fold changes between co‐ and monoculture illustrates the consistency between individual replicates of the treatments (Fig. S3).

### Comparative proteomics reveals fundamental changes in metabolism of *L. rostrata* when grown in coculture with *M. loti*

**Figure 1:**
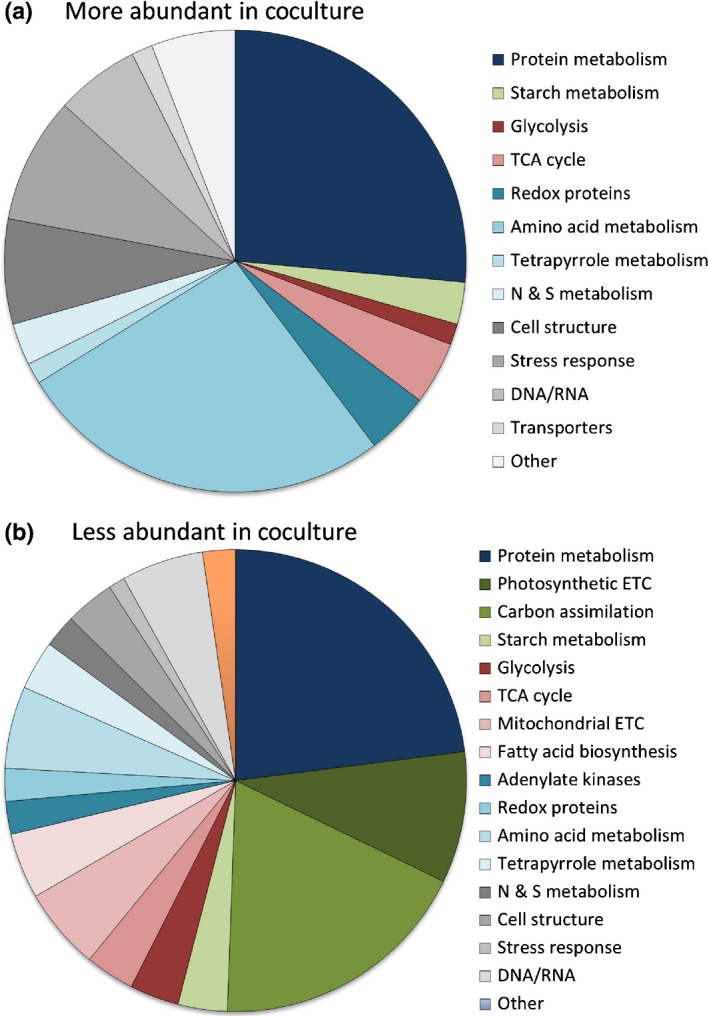
Overview of annotated functions ascribed to *Lobomonas rostrata* proteins. Proteins were extracted from 14 d cultures of *L. rostrata* grown in coculture with *Mesorhizobium loti*, or axenically supplemented with 100 ng l^−1^ cyanocobalamin (vitamin B_12_). (a) Relative proportions of each protein category determined by the quantitative isobaric tagging (iTRAQ) experiment to be more abundant in coculture (a total of 70 proteins). (b) Relative proportions of each protein category found in lower amounts (a total of 83 proteins). Proteins were categorized into major functional groups based on combined KEGG/Mercator (Kanehisa & Goto, 2000; Lohse *et al*., 2014) analysis. TCA, tricarboxylic acid; N & S, nitrogen & sulphur; ETC, electron transfer chain.

To establish how the metabolism of *L. rostrata* differed between the two treatments, we used a combined KEGG/Mercator‐based analysis (Kanehisa & Goto, 2000; Lohse *et al*., 2014) to assign function to the differentially expressed proteins, and to classify them into particular cellular processes and metabolic pathways (Table 1; Fig. 1). This analysis revealed that the metabolic processes most affected were those involved in protein metabolism (38 proteins, 24.8% of total), amino acid metabolism (23 proteins, 15%) and photosynthesis (24 proteins, 15.7%). Within the first category, which included those involved in general protein metabolism, translation, targeting and folding, about half were found in higher abundance in cocultured *L. rostrata* than in axenic culture, and half in lower amounts. Several chaperonins were elevated, along with cytosolic elongation and initiation factors. Four ribosomal proteins (S19, S20, L12 and L40) were in higher amounts, although another four (S2, S9, L3 and L8) were reduced. Strikingly, of the remaining 16 proteins of protein metabolism that were lower in cocultures, 15 were those of plastid protein synthesis, including several ribosomal proteins and elongation factors EF‐Tu and EF‐G.

**Table 1:** List of identified proteins altered in abundance in cocultures of *Lobomonas rostrata* vs axenic cultures

General function	Uniprot entry	Protein ID	Protein function	Fold‐change
Protein metabolism	A8ISZ1	EF‐3	Elongation factor EF‐3	2.69
	A8HX38	eEF1a1	Eukaryotic translation elongation factor 1 alpha 1 (EC 3.6.5.3)	2.55
	A8J597	RPL12	Ribosomal protein L12	2.42
	I2FKQ9	CPN60C	Mitochondrial chaperonin 60	2.41
	A8J8M9	RPS20	Ribosomal protein S20	2.18
	A8J7C8	KtRS	Lysine–tRNA ligase (EC 6.1.1.6) (Lysyl‐tRNA synthetase)	2.00
	Q66YD0	VIPP1	Chloroplast vesicle‐inducing protein in plastids 1	1.98
	A8J5Z0	RPP0	Acidic ribosomal protein P0	1.93
	A8I403	RPS19	Ribosomal protein S19	1.89
	A8HTK7	TBA1	PsbA translation factor	1.72
	A8JIB7	CPN60A	Chaperonin 60A	1.72
	P25840	HSP70	Heat shock 70 kDa protein	1.70
	A8ITH8	CPN60B2	Chaperonin 60B2	1.52
	A8JHX9	EFG2	Elongation factor 2 (EC 3.6.5.3)	1.51
	A8JCX9	RPL40	Ribosomal protein L40	1.44
	A8J9Q1	IPA1	Importin subunit alpha	1.33
	A8I232	EIF2G	Eukaryotic initiation factor	1.28
	A8JIE0	CPN20	Chaperonin 20	1.17
	A8IV59	TIIC22	22 kDa translocon at the inner membrane of chloroplasts (Fragment)	−1.14
	A8J7T7	CEP1	Cysteine endopeptidase	−1.16
	Q70DX8	S1PRPS1	Plastid ribosomal protein S1 (Ribosomal protein S1 homologue)	−1.27
	P17746	TUFA	Elongation factor Tu, chloroplastic (EF‐Tu)	−1.30
	E3SC57	RPL3	60S ribosomal protein L3 (Fragment)	−1.34
	Q5QEB2	EFTs	Elongation factor Ts	−1.34
	A8HME4	RPS2	Ribosomal protein S2	−1.37
	A8IA39	EFG1	Chloroplast elongation factor G (EC 3.6.5.3)	−1.41
	A8J282	CYN20	Peptidyl‐prolyl *cis‐trans* isomerase (EC 5.2.1.8) plastid	−1.41
	E3SC50	RPS9	40S ribosomal protein S9 (Fragment)	−1.42
	Q6Y683	RAP41	41 kDa ribosome‐associated protein (chloroplast)	−1.49
	Q6Y682	RAP38	38 kDa ribosome‐associated protein (Chloroplast stem‐loop‐binding protein)	−1.52
	Q8HTL1	RPL5	50S ribosomal protein L5, chloroplastic	−1.52
	A8IVK1	RPL8	Ribosomal protein L8	−1.53
	Q84U21	ERY2	50S ribosomal protein L22, chloroplastic	−1.60
	Q7Y258	PRPS5	Ribosomal protein S5 (plastid)	−1.61
	Q8GV23	NAB1	Nucleic acid binding protein (Putative nucleic acid binding protein)	−1.90
	A8I8Z4	PRPL1	Plastid ribosomal protein L1	−1.91
	A8IRU6	CYN38	Peptidyl‐prolyl *cis‐trans* isomerase, cyclophilin‐type (plastid)	−1.97
	O20032	RPS18	30S ribosomal protein S18, chloroplastic	−1.98
	A8HWS8	PRPL28	Plastid ribosomal protein L28	−2.75
Photosynthetic electron transfer	A8IV40	Fd	Apoferredoxin	−1.18
	A8I9I9	Re	Rieske ferredoxin	−1.19
	A8J0E4	PSBO	Oxygen‐evolving enhancer protein 1 of photosystem II	−1.21
	A8HXL8	ATPC	Chloroplast ATP synthase gamma chain	−1.25
	B7U1J0	ATPA	ATP synthase subunit alpha, chloroplastic (EC 3.6.3.14)	−1.32
	Q6PSL4	HYDG	Fe‐hydrogenase assembly protein (Hydrogenase assembly factor)	−1.36
	A8J6Y8	FNR2	Ferredoxin–NADP reductase (EC 1.18.1.2)	−1.63
	P53991	PETH	Ferredoxin–NADP reductase, chloroplastic (FNR) (EC 1.18.1.2)	−1.66
Carbon assimilation	A8IVM9	GCSP	Glycine cleavage system, P protein (EC 1.4.4.2)	−1.15
	Q6SA05	RCA1	Rubisco activase	−1.23
	P23489	RA	Ribulose bisphosphate carboxylase/oxygenase activase, chloroplastic	−1.36
	Q0ZAZ1	GDH	Glycolate dehydrogenase	−1.37
	A8HP84	GAP3	Glyceraldehyde‐3‐phosphate dehydrogenase (EC 1.2.1.12) (EC 4.3.2.1)	−1.47
	A8IPI7	HPR1	Hydroxypyruvate reductase	−1.51
	A8IRK4	SEBP1	Sedoheptulose‐1,7‐bisphosphatase (EC 3.1.3.37)	−1.53
	A8IKW6	RPE1	Ribulose‐phosphate 3‐epimerase (EC 5.1.3.1)	−1.57
	A8I5A0	CPLD45	Predicted protein	−1.58
	A8IKQ0	FBP1	Fructose‐1,6‐bisphosphatase (EC 3.1.3.11)	−1.65
	Q42690	FBA1	Fructose‐bisphosphate aldolase 1, chloroplastic (EC 4.1.2.13)	−1.71
	A8IRQ1	RPI1	Ribose‐5‐phosphate isomerase (EC 5.3.1.6)	−1.72
	Q75NZ2	LCIB	Low‐CO_2_ inducible protein LCIB	−1.77
	A8JC04	PGK1	Phosphoglycerate kinase (EC 2.7.2.3)	−1.81
	P93109	CA2	Beta‐carbonic anhydrase	−1.95
	Q39589	BETA‐CA1	Carbonic anhydrase	−2.91
Starch metabolism	Q2VA40	PHOA	Phosphorylase (EC 2.4.1.1)	1.48
	Q2VA41	PHOB	Phosphorylase (EC 2.4.1.1)	1.42
	A8HS14	STA1	ADP‐glucose pyrophosphorylase (EC 2.7.7.27)	−1.23
	Q9LLL6	STA6	ADP‐glucose pyrophosphorylase (EC 2.7.7.27)	−1.34
	O64926	SS	Soluble starch synthase (EC 2.4.1.21)	−1.81
Glycolysis	A8JHR9	GAP1A	Glyceraldehyde 3‐phosphate dehydrogenase, dominant splicing variant (EC 1.2.1.12)	3.01
	A8 IE23	PGI1	Glucose‐6‐phosphate isomerase (EC 5.3.1.9)	−1.22
	A8IVR6	PYK2	Pyruvate kinase (EC 2.7.1.40)	−1.70
	A8JC04	PGK1	Phosphoglycerate kinase (EC 2.7.2.3)	−1.81
TCA cycle	A8J2S0	CIS2	Citrate synthase (EC 2.3.3.1)	1.82
	A8JHC9	CIS1	Citrate synthase	1.49
	A8HMQ1	ACH1	Aconitate hydratase (EC 4.2.1.3)	1.41
	Q9FNS5	MDH5	NADP‐Malate dehydrogenase (EC 1.1.1.82)	−1.27
	A8HPL8	DLD2	Dihydrolipoamide dehydrogenase (EC 1.8.1.4)	−1.58
	A8J7F6	DLA2	Dihydrolipoamide acetyltransferase (EC 2.3.1.12)	−1.59
Mitochondrial electron transfer chain	A8JDV9	ATP3	F1F0 ATP synthase gamma subunit	−1.17
	Q96550	ATPA	ATP synthase subunit alpha	−1.32
	Q6V9B1	NUOS8	NADH:ubiquinone oxidoreductase subunit 8 (EC 1.6.5.3)	−1.32
	A8IVJ7	NUOS1	NADH:ubiquinone oxidoreductase 76 kDa subunit	−1.49
	Q6V9A8	NUO7	NADH:ubiquinone oxidoreductase 49 kD subunit (EC 1.6.5.3)	−1.77
Fatty acid biosynthesis	A8HPL8	DLD2*	Dihydrolipoamide dehydrogenase (EC 1.8.1.4)	−1.58
	A8J7F6	DLA2*	Dihydrolipoamide acetyltransferase (EC 2.3.1.12)	−1.59
	A8JFI7	EAR	enoyl ACP reductase	−1.92
	A8JGF4	BC	Biotin carboxylase, acetyl‐CoA carboxylase component	−1.98
Tetrapyrrole metabolism	A8I980	ALAD	Delta‐aminolevulinic acid dehydratase (EC 4.2.1.24)	1.41
	A8J7H3	GSA	Glutamate‐1‐semialdehyde aminotransferase (EC 5.4.3.8)	−1.23
	A8HNE8	CHLP	Geranylgeranyl reductase (EC 1.3.1.‐)	−1.33
	A8HPJ2	POR	Light‐dependent protochlorophyllide reductase (EC 1.3.1.33)	−1.59
Amino acid metabolism	A8IH03	PSAT	Phosphoserine aminotransferase (EC 2.6.1.52)	3.11
	A8J786	HIS7	Imidazole glycerol phosphate synthase (Fragment)	2.95
	A8J2X6	AGGPR	*N*‐acetyl‐gamma‐glutamyl‐phosphate reductase	2.29
	A8HXS9	LEU2	Isopropylmalate synthase (EC 2.3.3.13) (Fragment)	2.18
	A8IXE0	SAHH	S‐Adenosylhomocysteine hydrolase (EC 3.3.1.1)	1.99
	A8JFR4	OTA1	Ornithine transaminase (EC 2.6.1.13)	1.95
	A8JG03	LEU1	Isopropylmalate dehydratase, large subunit	1.94
	A8J979	MCCA	Methylcrotonoyl‐CoA carboxylase alpha subunit	1.85
	A8I826	HSK1	Homoserine kinase (EC 2.7.1.39)	1.81
	A8IMY5	ANS1	Anthranilate synthase, alpha subunit (Fragment)	1.70
	A8J173	ASD1	Aspartate semialdehyde dehydrogenase	1.62
	A8J2Z6	ChS1	Chorismate synthase (EC 4.2.3.5)	1.47
	A8IX80	AAD1	Acetohydroxyacid dehydratase	1.46
	A8ISB0	OASTL4	O‐acetylserine sulphydrolase; cysteine synthase (EC 2.5.1.47)	1.42
	A8J355	CGS1	Cystathionine gamma‐synthase	1.42
	A8J3D3	HIS5	Histidinol phosphate aminotransferase	1.41
	A8ITU7	PGD1	D‐3‐phosphoglycerate dehydrogenase (EC 1.1.1.95)	1.37
	O22547	ALAS	Acetolactate synthase	1.20
	A8JIR0	CPS1	Carbamoyl phosphate synthase, large subunit (EC 6.3.5.5)	−1.17
	P22675	ARG7	Argininosuccinate lyase (ASAL) (EC 4.3.2.1) (Arginosuccinase)	−1.26
	A8I305	GLN1	Glutamine synthetase (EC 6.3.1.2)	−1.43
	A8JBL3	NAGS	*N*‐acetylglutamate synthase (Fragment)	−1.49
Other	A8IJL3	MEP‐S	2‐C‐methyl‐d‐erythritol 2,4‐cyclodiphosphate synthase (EC 4.6.1.12)	2.02
	A8J841	THICb	Hydroxymethylpyrimidine phosphate synthase	2.00
	A8HMC0	CRT2	Calreticulin 2, calcium‐binding protein	1.53
	Q8LRU1	FER1	Pre‐apoferritin	1.41
Kinases	A8JDN2	ADK3	Adenylate kinase 3 (EC 2.7.4.3)	−1.97
	A8JH12	–	Nucleoside diphosphate kinase	−1.97
Redox proteins	O22472	CAT	Catalase (EC 1.11.1.6)	2.16
	A8HQT1	PDI2	Protein disulfide isomerase	1.54
	A8IA77	GSH1	Gamma‐glutamylcysteine synthetase (EC 6.3.2.2)	1.54
	A8IVV9		Flavodoxin‐like protein (Fragment)	−1.16
	A8IWK2	–	Ferredoxin thioredoxin reductase, catalytic chain	−2.14
N & S metabolism	A8JHB4	GSF1	Ferredoxin‐dependent glutamate synthase	1.29
	A8IXF1	ATS1	ATP‐sulfurylase	1.15
	A8J6A7	MET16	Adenylylphosphosulfate reductase	−1.27
	A8JGD1	GDH2	Glutamate dehydrogenase	−1.36
Cell structure/organization	A8JB85	APG8	Autophagy protein	3.84
	A8HW56	CDC48	Flagellar associated protein (EC 3.6.1.3)	2.08
	A8J614	FAP42	Flagellar associated protein, adenylate/guanylate kinase‐like protein	1.80
	A8IFL6	FAP262	Flagellar associated protein	1.70
	A8JEQ8	FAP79	Flagellar associated protein	1.31
	A8JAV1	IDA5	Actin	−1.31
	P09205	TUBA2	Tubulin alpha‐2 chain	−1.90
	A8IXZ0	TUB1	Beta tubulin 1 (Beta tubulin 2)	−2.11
Stress response	Q39603	HSP70B	Heat shock protein 70B	2.06
	A8IZU0	HSP70C	Heat shock protein 70C	1.77
	P25840	HSP70	Heat shock 70 kDa protein	1.70
	A8I7T1	HSP90B	Heat shock protein 90B (Fragment)	1.62
	A8I972	CLPB3	ClpB chaperone, Hsp100 family	1.61
	A8HYV3	HSP70B	Heat shock protein 70B	1.32
DNA and RNA metabolism	A8JFP3	–	Histone methyltransferase	1.34
	Q1G2Y1	PNP	Chloroplast polyribonucleotide phosphorylase	1.30
	A8IZS5	–	Glycine‐rich RNA‐binding protein	1.23
	A8I0F5	ORC1	Origin recognition complex subunit 1	1.21
	A8JCT1	HBV1	Histone H2B	−1.77
	A8HWY2	HTR14	Histone H3 (Fragment)	−1.88
	A8HVA3	HFO4	Histone H4	−1.91
	A8JIN9	HFO43	Histone H4	−1.92
	A8HRZ9	H2A	Histone H2A	−1.95
Transporters	A8JGB0	–	Arsenite translocating ATPase‐like protein (Fragment)	1.44
	A8I164	ATPVA1	Vacuolar ATP synthase, subunit A	−1.18
	A8I268	HLA3	ABC transporter	−2.40

Peptides from the quantitative isobaric tagging (iTRAQ) experiment were identified by comparison with the *Chlamydomonas reinhardtii* proteome (indicated by the Uniprot ID). A total of 70 proteins were found to be significantly (*P *<* *0.05) more abundant in the cocultures with *Mesorhizobium loti* (positive values), and 83 were less abundant (negative values). Identified proteins are grouped into functional categories as in Fig. 1, ranked according to fold‐change. Data are available via ProteomeXchange (PXD005046). *Also involved in the tricarboxylic acid (TCA) cycle.

**Figure 2:**
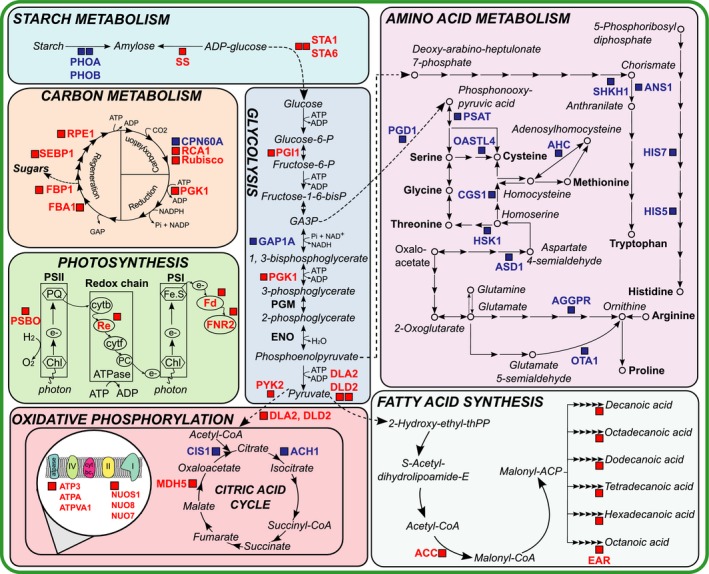
Schematic diagram summarizing changes in primary metabolic enzymes of *Lobomonas rostrata* in coculture vs in axenic culture with B_12_ supplementation. Proteins found in higher amounts in coculture are indicated in blue, whereas those that are less abundant are shown in red. Abbreviations are provided in Table 1.

This effect on chloroplast biology was further reflected in the substantial number of proteins associated with both the light‐dependent reactions of photosynthesis and CO_2_ fixation, which were much lower in the coculture; none were elevated (Table 1; red boxes in Fig. 2). There is reduced abundance of subunits of the chloroplast ATP synthase alongside those from the oxygen‐evolving complex of PSII, cytochrome b_6_f complex (Rieske Fe.S protein, Re), and soluble electron carriers ferredoxin (Fd) and ferredoxin‐NADP reductase (FNR). Several enzymes in the Calvin cycle were also in lower abundance, including RuBisCO activase (RCA1) and two enzymes involved in photorespiration, hydropyruvate reductase and glycolate dehydrogenase (Chauvin *et al*., 2008). In addition, there was a decrease in two carbonic anhydrases, which catalyse conversion of carbon dioxide to bicarbonate, one of which showed the greatest log_2_‐fold change (−2.91) of all detected proteins.

Subunits of soluble starch synthase and ADP‐glucose pyrophosphorylase (STA1, STA6), both involved in starch synthesis, were less abundant in the coculture, whereas starch phosphorylase, important for starch remobilization, was higher. Similarly, enzymes of glycolysis and the tricarboxylic acid (TCA) cycle also showed a mixture of higher and lower abundance, but all the subunits of the mitochondrial electron transfer chain that were detected (three subunits of NADH:ubiquinone oxidoreductase and two subunits of mitochondrial ATP synthase) were reduced in cocultures compared with axenic *L. rostrata*, as were enzymes involved in fatty acid biosynthesis, enoyl ACP reductase and the biotin carboxylase subunit of the acetyl‐CoA carboxylase enzyme complex (Ohlrogge & Jaworski, 1997), and three out of four enzymes of tetrapyrrole biosynthesis, including two of the Chl branch, light‐dependent protochlorophyllide reductase and geranylgeranyl PP reductase. The impression is that in *L. rostrata* grown with *M. loti* there is a reduction in biosynthesis of the photosynthetic apparatus and activity.

By contrast, many enzymes of amino acid metabolism were in higher abundance in cocultured *L. rostrata* compared with monocultures, with a total of 18 enzymes involved in both the degradation and biosynthesis of amino acids being elevated (Fig. 2). Among them were anthranilate synthase, involved in the tryptophan biosynthesis pathway (Liu *et al*., 2010), cysteine synthase (*O*‐acetylserine sulphydrolase, OASTL4), the final enzyme of the synthesis of cysteine (Tai *et al*., 2001), methylcrotonoyl‐CoA carboxylase, used in the degradation of leucine to acetoacetate (Song *et al*., 1994), and S‐adenosylhomocysteine hydrolase, involved in the methylation cycle of C1 metabolism (Palmer & Abeles, 1979). Just two proteins involved in amino acid metabolism were lower in cocultured *L. rostrata*,* N*‐acetylglutamate kinase and argininosuccinate lysase, enzymes of the urea cycle and important for production of fumarate for the TCA cycle (Shargool *et al*., 1988). Ferredoxin‐glutamate synthase (Fd‐GOGAT), involved in nitrate assimilation, was also elevated in cocultures, as was ATP‐sulphurylase involved in S‐assimilation, although adenylylphosphosulfate reductase was reduced. Two other enzymes of biosynthetic pathways were elevated: 2‐C‐methyl‐d‐erythritol 2,4‐cyclodiphosphate synthase, an enzyme of the nonmevalonate pathway for the production of the isoprenoid precursor isopentenyl‐pyrophosphate (Eisenreich *et al*., 2001); and THIC, encoding an enzyme of *de novo* thiamine biosynthesis.

Of particular note in the other functional categories was the higher abundance in the cocultures of stress‐response related proteins, comprising six proteins annotated as heat shock proteins (HSPs): four annotated as members of the HSP70 family, the others as a chloroplast‐targeted HSP101 homologue and an HSP90‐like protein. Also elevated were four flagellar‐associated proteins and autophagy protein (APG8), the latter being the most highly altered relative to axenic *L. rostrata* (3.84). And whilst five histones are lower in abundance in coculture, histone methylase and three other enzymes of DNA metabolism were found in higher amounts.

**Figure 3:**
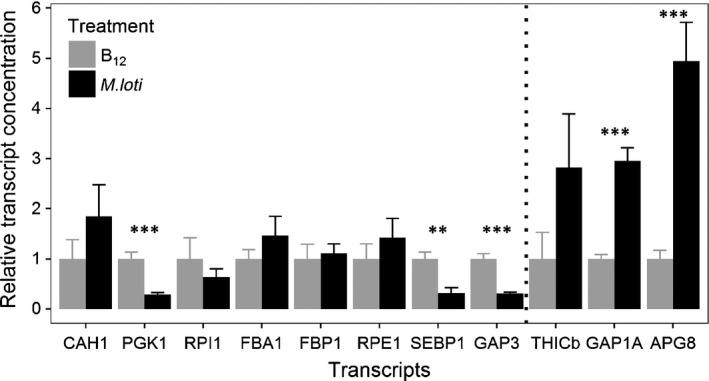
Reverse transcriptase‐quantitative polymerase chain reaction (RT‐qPCR) measurements of transcript abundance. Transcripts of genes encoding enzymes of photosynthesis that were found in lower abundance in cocultures, plus three proteins highly elevated in cocultures were quantified using cDNA prepared from RNA extracted from 14‐d‐old cultures. The transcripts labelled on the *x*‐axis are in ascending order of protein fold‐change measured by the quantitative isobaric tagging (iTRAQ) experiment. The vertical dashed line separates proteins that are lower in abundance in coculture (left) from those that are higher (right). Transcript abundance was normalized against three housekeeping genes (EIF4A, UBQ, RACK1) and then levels in cocultures with *Mesorhizobium loti* (black columns) are shown relative to the level in axenic *Lobomonas rostrata* cultures (grey columns) set as 1. Error bars, + SE; *n *≥ 3; *P*‐value (Student's *t*‐test): **, *P *<* *0.01; ***, *P *<* *0.001. Abbreviations are as in Table 1.

Given the observed reduction in amounts of almost all the proteins of the Calvin cycle, we decided to determine if this was a result of changes in transcript expression levels. We therefore carried out RT‐qPCR using as template RNA extracted from cultures grown in conditions identical to those used for proteomics analysis. We were able to identify sequences for eight proteins involved in carbon fixation by searching an RNAseq dataset from *L. rostrata* (U.J. Kudahl *et al*., unpublished) using the *C. reinhardtii* homologues, to allow design of appropriate PCR primers. We also found *L. rostrata* homologues for three of the most elevated proteins, *THIC*,* APG8*, and *GAP1A*, encoding cytosolic glyceraldehyde 3‐phosphate dehydrogenase. Fig. 3 shows the results in order of the fold‐abundance detected in the iTRAQ experiment, with those lower in coculture than in monoculture on the left, and those higher on the right. In general, the trend in the transcript abundances mirrored that of the proteins, with significant (*P* < 0.001) up‐regulation of *GAP1A* and *APG8* in coculture (*c*. three‐ and fivefold, respectively). The thiamine biosynthesis enzyme *THIC* was also elevated, although this was not statistically significant. Similarly, there was significant (*P*‐value < 0.001) down‐regulation of phosphoglycerate kinase 1(*PGK1*) and chloroplast glyceraldehyde 3‐phosphate dehydrogenase 3 (*GAP3*), as well as sedoheptulose‐1,7‐bisphosphatase (*SEBP1*;* P *<* *0.01). The transcript abundances for the other photosynthetic genes were unaltered or slightly higher in coculture, but not significantly. It should be noted that the *CAH1* gene, encoding a carbonic anhydrase, may encode a different isoform to the proteins detected in the iTRAQ experiment.

### Photosynthetic ETR is reduced in coculture with *M. loti*

**Figure 4:**
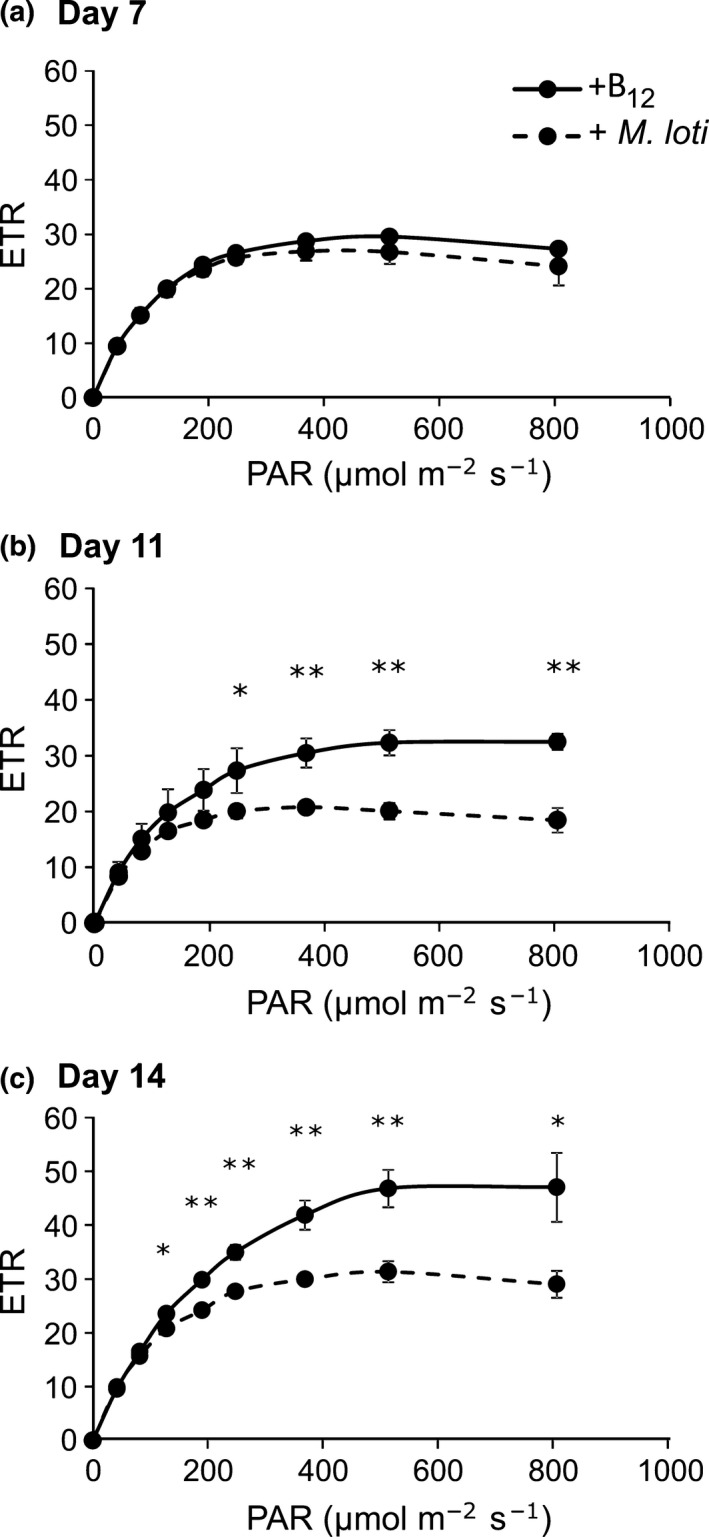
Photosynthetic capacity of *Lobomonas rostrata* cultures. Pulse amplitude modulation (PAM) measurements of the electron transfer rate (ETR) were made to estimate overall photosynthetic capacity. The solid line shows data from cultures grown axenically with 100 ng l^−1^ cyanocobalamin, while dashed lines show the ETR from cocultures with *Mesorhizobium loti*. Rates were measured in samples taken from cultures at (a) day 7, (b) day 11, and (c) day 14 after inoculation. Cells were diluted to equivalent cell densities (2.5 × 10^6^ cells ml^−1^). Data are the mean of three replicates ± SEM. Significant differences in ETR between treatments (two‐sample *t*‐test): *, *P *≤* *0.05; **, *P *≤* *0.01.

Our observation that several proteins involved in both the light reactions of photosynthesis and carbon dioxide fixation were less abundant in *L. rostrata* cells in coculture with *M. loti* prompted us to investigate whether these altered protein abundances impacted photosynthetic activities of cells in the symbiotic vs axenic treatments. Accordingly, we made photophysiological measurements of cells in the two treatments using PAM fluorimetry. Quantification of the quantum yield of PSII and photosynthetic ETR provides information concerning the overall photosynthetic performance of a culture. *L. rostrata* cultures, with either 100 ng l^−1^ B_12_ or *M. loti*, were grown in identical conditions to those described for the proteomics experiment. Measurements of ETR were made at 7, 11 and 14 d after inoculation into fresh medium, corresponding to three different growth stages of the culture (Fig. S2). As can be seen in Fig. 4, at 7 d, there is little difference in ETR, but at both days 11 and 14, it was significantly lower in cells grown in the presence of *M. loti*, with the effect becoming more pronounced, so that at day 14 (corresponding to samples used for the proteomics analysis) the rate was only *c*. 60% of that in axenic cultures.

### *L. rostrata* cells grown in coculture with *M. loti* have higher amounts of amino acids and total protein

**Figure 5:**
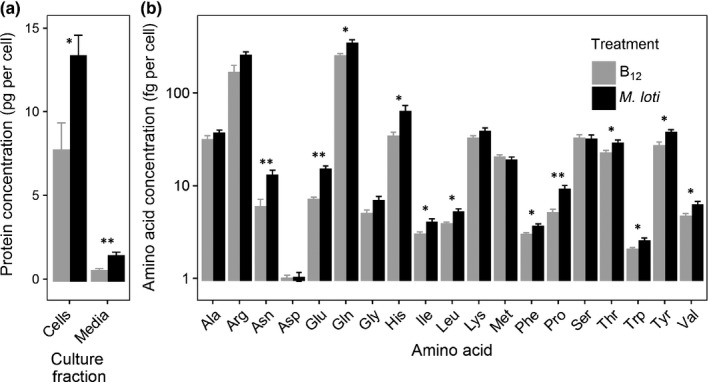
Protein and amino acid content of *Lobomonas rostrata* cultures. (a) Total protein from the cell pellet or media of 14‐d‐old cultures of axenic *L. rostrata* or of *L. rostrata* in coculture with *Mesorhizobium loti*. Protein is expressed in pg per algal cell relative to a standard of BSA. (b) Amino acids from the cell pellet on day 14 were separated by high‐performance liquid chromatography (HPLC) and quantified by MS. Amino acids are labelled on the *x*‐axis in alphabetical order, and quantities are expressed in fg per algal cell on the *y*‐axis. Axenic *L. rostrata* + 100 ng l^−1^ B_12_, grey columns; *L. rostrata* + *M. loti*, black columns. Error bars, + SE; *n *=* *4; *P*‐value (Student's *t*‐test): *, *P *<* *0.05; **, *P *<* *0.01.

The other major group of proteins that were altered were enzymes of amino acid biosynthesis. Accordingly we measured total protein in the cultures and found that for both the cell pellet and the media, the amount in the coculture was approximately double that in the axenic cultures (Fig. 5a). Whilst the former contain bacterial cells in addition, the estimated contribution of the bacterial cells to the total protein is probably < 10% (see earlier). We also determined the profile of free amino acids in the cells using HPLC‐MS (Fig. 5b; note the log scale for the *y*‐axis; Table S2). The majority of the 15 protein amino acids that could be identified by this method were elevated in coculture, with up to twofold changes seen for asparagine (Asn), glutamate (Glu) and histidine (His). The exceptions were methionine and serine, for which slight decreases were observed. Given that methionine is the product of the enzyme in *L. rostrata* that requires B_12_ (cobalamin‐dependent methionine synthase, METH), this might indicate B_12_ limitation, a conclusion supported by elevation of SAHH in coculture, and the observation that addition of B_12_ to the media of cocultures enhances growth of *L. rostrata* (Kazamia *et al*., 2012).

## Discussion

In this study we applied quantitative iTRAQ proteomics to understand the metabolic differences in *L. rostrata* cells when grown in media supplemented with vitamin B_12_ as compared with cultures in which B_12_ is provided by *M. loti* cells. *L. rostrata* does not have a sequenced genome, and therefore its proteome is unknown, and the presence of bacterial cells within the coculture samples also complicated the analysis. A series of preliminary experiments were undertaken, an approach that is essential for these sorts of coculture, cross‐species proteomics experiments. Through these analyses we demonstrated that *C. reinhardtii* would provide a suitable reference database by a 1% PSM rate to *L. rostrata* peptides; that a low (0.31%) shared tryptic peptide (length of six to 16 amino acids) between *C. reinhardtii* and *M. loti* minimized interference by shared peptides; and that the *c*. 14‐fold less bacterial protein compared with algal protein in the coculture would not be detrimental to the overall algal protein numbers confidently identified and relatively quantified using iTRAQ. The value of these preliminary experiments was successfully demonstrated during the iTRAQ experiment, where we were able to identify 588 *L. rostrata* proteins with at least one high‐confidence peptide hit. Protein fold changes were comparable to previous iTRAQ studies, where relative values are significantly lower than usually seen for transcript‐level quantifications. To avoid using arbitrary thresholds for highlighting differentially expressed proteins, stringent quantitative statistics were applied (Noirel *et al*., 2011) and revealed 153 proteins that were significantly differentially expressed (*P*‐value < 0.05). (Table 1; Fig. 1). Furthermore, whilst transcript expression may not necessarily reflect protein abundance, qRT‐PCR analysis showed that transcript and protein expression were correlated in the two treatments for several of the genes we sampled (Fig. 3), providing further verification of our approach.

Inspection of the classes of protein that were altered revealed a higher abundance of proteins related to amino acid biosynthesis (Fig. 2a), and this was accompanied by modest but significant increases in several protein amino acids in the algal cells (Fig. 5b). *M. loti* is dependent on *L. rostrata* for fixed carbon in the cocultures, but the specific compound (or compounds) that the alga supplies is currently unknown. It is possible, therefore, that amino acids are involved, as has been documented for several other symbiotic interactions. For example, growth of the heterotrophic bacterium *Shewanella* W3‐18‐1 with the cyanobacterium *Synechococcus* sp. PCC 7002 modulates amino acid metabolism in *Shewanella* (Beliaev *et al*., 2014). A reduction in the expression of genes involved in alanine and methionine biosynthesis in this bacterium appear to have resulted from enhanced excretion of these amino acids by the cyanobacterial partner, indicative of metabolite exchange. Perhaps the best‐known example of amino acids exchange is provided by legume–rhizobial interactions, where the nitrogen fixed by the symbiotic bacteria is transferred to their legume partners in the form of ammonium, some of which is metabolized by the plant and returned to the bacteria in the form of amino acids in order to support their growth (Lodwig *et al*., 2003). There are further similarities between proteins significantly up‐regulated in cocultured *L. rostrata* and those in the nodule symbiosome, the membrane bordering the nitrogen‐fixing root nodule. These include a 60 kDa chaperonin, a homologue of that found in symbiosomes of *Glycine max* (Panter *et al*., 2000), HSP 70 found in symbiosomes of *Lotus japonicus* and *Medicago truncatula* (Wienkoop & Saalbach, 2003; Catalano *et al*., 2004), and calreticulin, also found in *L. japonicus* (Wienkoop & Saalbach, 2003). The canonical function of chaperonins and HSPs is in assisting correct protein folding and preventing protein aggregation in cells undergoing some form of stress (Saibil, 2013). Other proteins up‐regulated in cocultured *L. rostrata* that are also found in the symbiosome membrane are D‐3‐phosphoglycerate dehydrogenase involved in amino acid biosynthesis (Wienkoop & Saalbach, 2003), citrate synthase (Saalbach *et al*., 2002), cysteine synthase (Wienkoop & Saalbach, 2003) and lysine‐tRNA ligase (Catalano *et al*., 2004). The prokaryotic isoforms of some of these proteins have also been found to be present in the symbiosome space (the area between the plant and bacterial cell membranes in the symbiosome) rather than the membrane, indicating that they share a common symbiotic function between kingdoms (Emerich & Krishnan, 2014). The exact function these proteins have acquired in the symbiosome is still largely unknown. In other symbiotic systems, mostly parasitic, the prokaryotic isoforms of these proteins are thought to be important in the cell‐to‐cell adhesion of symbiont and host and it could be that the same is true for the eukaryotic isoforms (Copley, 2012), although it is known that cell‐to‐cell contact is not an obligate requirement for our *L. rostrata* and *M. loti* system (Kazamia *et al*., 2012).

Perhaps the most striking feature of *L. rostrata* grown in coculture with *M. loti* is that there is an overall reduction in many chloroplast proteins. First, proteins involved in chloroplast protein synthesis and import are much lower in coculture, whereas several of the cytosolic equivalents are elevated. Correlating with this observation is a reduction in two chloroplast‐encoded subunits of the ATP synthase. At the same time, many components of the nucleus‐encoded, light‐dependent reactions of photosynthesis (Fig. 2), including the Rieske Fe.S subunit (Re) of the cytochrome complex, ferredoxin (Fd), and Fd‐NADP reductase (FNR), and virtually all of the Calvin cycle enzymes are also found in lower amounts in the coculture cells. Measurement of the ETR confirmed that the photosynthetic capacity of *L. rostrata* cells grown with *M. loti* in coculture is significantly lower than that in cells grown in axenic culture (Fig. 4). This is in contrast to a transcriptomics analysis of the cyanobacterium *Prochlorococcus* NATL2A grown in coculture with the heterotrophic bacterium *Alteromonas macleodii* MIT 1002, where a notable enhancement of genes encoding photosynthetic apparatus, including subunits of PSI and Chl biosynthesis proteins, was seen in the cyanobacterium. The profound impact that interactions can have on growth and Chl fluorescence parameters has also been highlighted by a systematic physiological study that screened a library of hundreds of heterotrophic marine bacteria in pairwise cocultures with different *Prochlorococcus* ecotypes (Sher *et al*., 2011). In general, this study identified that ‘enhancing interactions’, whereby the presence of heterotrophic bacteria enhanced cyanobacterial growth rate and/or Chl fluorescence, were more common than the other way round. However, in this work, the treatment conditions were such that all the necessary nutrients for growth were present in the medium (in this case Pro99 medium supplemented with pyruvate, acetate, lactate, glycerol, alongside vitamins) even in the presence of the bacterium, which is in contrast to our setup in which the coculture treatment lacked a bacterial carbon source, alongside a vital micronutrient necessary for *L. rostrata* growth. Thus, in a scenario in which growth of both microbial partners is absolutely dependent of the presence of the other, a fine balance between mutualism and competition appears to be in play. Whilst *L. rostrata* growth (and photosynthetic output) is supported by a B_12_‐synthesizing bacterium, it is nevertheless constrained compared with growth in media where all necessary nutrients are in adequate supply. There may also be a degree of competition between the two species for other nutrients in the media (such as nitrogen and trace elements) and the cocultured alga may dedicate efforts to producing resource acquisition proteins rather than photosynthesis‐related proteins. In fact, in the closely related algae *C. reinhardtii*, it is well known that photosynthetic gene expression declines during early nutrient deprivation (Zhang *et al*., 2004; Schmollinger *et al*., 2014), which has been suggested to be a result in part of accumulation of transcripts for *rls1*, a putative transcriptional repressor of nuclear‐encoded chloroplast proteins (Nedelcu, 2009). This competition may also provide another explanation for the up‐regulation of amino acid biosynthesis enzymes in cocultured *L. rostrata*, the cocultured alga requiring more resource acquisition machinery rather than providing amino acids to its symbiont (Arrigo, 2005).

A previous study of a mutualistic coculture based on B_12_ exchange, in this case between the marine diatom *Thalassiosira pseudonana* and a roseobacter *Ruegeria pomeroyi* DSS‐3, looked at transcript‐level changes and identified that the likely compound supplied by the alga was the sulphur‐containing compound 2,3‐dihydroxypropane‐1‐sulfonate (Durham *et al*., 2015). They found no evidence of alterations in transcripts for enzymes of the photosynthetic machinery, nor any change in photosynthetic activity. By contrast, the results of our study at the protein level, which is a more direct indication of cellular activity, suggest, perhaps counterintuitively, that a photosynthetic organism grown mutualistically with a bacterium does not increase its photosynthetic activity to support both organisms. Instead, the photosynthetic capacity of the alga is probably more constrained by its own nutrient status than by the demands of its partner. In this system, where the nutrient (B_12_) status of the alga is effectively set by the bacterium, and vice versa (in terms of carbon supply), there appears to be a tradeoff in terms of the algal response. The striking impact that cohabiting bacteria can have on algal physiology, evolution and metabolism is becoming increasingly recognized. Algae and bacteria are among the most abundant organisms on the planet, and together their metabolism impacts global nutrient cycling and energy flow. Thus, enhancing our knowledge of the molecular basis of algal–bacterial interactions is critical to establishing the fundamental core principles governing microbial community function in the aquatic biosphere.

## Author contributions

J.P., K.E.H., P.C.W. and A.G.S. conceived the project and designed the research; K.E.H., M.B.C., J.L., U.J.K, D.A.R., E.V.T., F.B., D.L.S. and N.S. carried out the research; K.E.H., M.B.C, U.J.K, F.B., N.S., J.P. and A.G.S. carried out the data analysis and interpretation; K.E.H., M.B.C., J.P. and A.G.S. wrote the manuscript, with input from all authors. All authors read and approved the final manuscript.

## Supporting information

Please note: Wiley Blackwell are not responsible for the content or functionality of any Supporting Information supplied by the authors. Any queries (other than missing material) should be directed to the *New Phytologist* Central Office.

**Fig. S1** Proteomics work flow.
**Fig. S2** Growth over time of *L. rostrata* grown in axenic culture with B_12_ supplementation (100 ng l^−1^) versus in coculture with *M. loti*.
**Fig. S3** Heat map of proteins identified from iTRAQ analysis.
**Table S1** List of primers used for reverse transcriptase‐quantitative polymerase chain reaction (RT‐qPCR)
**Table S2** Optimized values for MS analysis of amino acids
**Methods S1** Amino acid analysis.
