# Supplementary material for: Quantitative proteomics of a B12‐dependent alga grown in coculture with bacteria reveals metabolic tradeoffs required for mutualism
Source: New Phytol. 2017 Oct 16;217(2):599–612. doi: 10.1111/nph.14832 (PMC5765456; doi:10.1111/nph.14832)
Supplement: Supplementary file 1 — Fig. S1 Proteomics work flow. Fig. S2 Growth over time of L. rostrata grown in axenic culture with B12 supplementation (100 ng l−1) versus in coculture with M. loti. Fig. S3 Heat map of proteins identified from iTRAQ analysis. Table S1 List of primers used for reverse transcriptase‐quantitative polymerase chain reaction (RT‐qPCR) Table S2 Optimized values for MS analysis of amino acids Methods S1 Amino acid analysis. [file NPH-217-599-s001.pdf]

New Phytologist **Supporting Information**

Article title: **Quantitative proteomics of a B_{12}-dependent alga grown in co-culture with bacteria reveals metabolic trade-offs required for mutualism**

Authors: Katherine E. Helliwell, Jagroop Pandhal, Matthew B. Cooper, Joseph Longworth, Ulrich Johan Kudahl, David A. Russo, Eleanor V. Tomsett, Freddy Bunbury, Deborah L. Salmon, Nicholas Smirnoff, Phillip C. Wright, Alison G. Smith

Article acceptance date: 31 August 2017

The following Supporting Information is available for this article:

Method S1: Amino acid analysis

Fig. S1 Proteomics workflow

Fig. S2 Growth curve for *L. rostrata*

Fig. S3 Heat map of proteins identified in different conditions

Table S1 List of primer sequences

Table S2 Optimised values for Mass-spectroscopic analysis of amino acids

Methods S1

QQQ LC-MS Amino Acid Analysis

Approximately 5mg of freeze-dried sample was extracted in 400 µl of acetonitrile (LC-MS grade, Sigma-Aldrich), vortexing to ensure the pellet was in fine suspension. Samples were sonicated for 15 minutes, vortexed, and centrifuged at 13K rpm, 4°C, for 20 min in a Beckman-Coulter benchtop centrifuge. The supernatant was filtered through a 0.2 µM syringe tip filter (Phenomenex, UK) into clean 1.5ml centrifuge tube. This extraction process was repeated using 400ul 20% Methanol (LC-MS grade) spiked with an internal standard containing stable isotope-labelled amino acids (L-amino acid mix (Sigma-Aldrich, Co., St. Louis, MO, USA)) to give a concentration of 0.5 µM per sample. The supernatants combined for each sample. An AccQ•Tag Ultra derivatization kit (Waters Corporation, Milford, MA, USA) was used for derivatizing 10µl of extracted sample. The samples were immediately vortexed followed by incubation for 15 min at 55°C prior to analysis.

HPLC-ESI-MS/MS quantitative analysis of the amino acids was performed using an Agilent 6420B triple quadrupole (QQQ) mass spectrometer (Technologies, Palo Alto, USA) hyphenated to a 1200 series Rapid Resolution HPLC system. 5 µl of derivatised sample extract was loaded onto an Eclipse Plus C18 (3.5 µm, 2.1 x 150 mm) reverse phase analytical column (Agilent Technologies, Palo Alto, USA) with an Eclipse Plus C18 (1.8 µm, 2.1x5mm) guard (Agilent Technologies, Palo Alto, USA). For detection using positive ion mode, mobile phase A comprised of 95% LC-MS grade H2O, with 0.1 % formic acid and 1 mM ammonium formate, and mobile phase B was 95% acetonitrile (LC-MS grade) with 0.1 % formic acid. The following gradient was used: 0 min – 0% B; 16 min – 20% B; 20 min – 100% B; 22 min – 100% B; 23 min – 0% B followed by 3 min re-equilibration time. The flow rate was 0.25 ml min^{-1} and the column temperature was held at 35 °C for the duration. The QQQ source conditions for electrospray ionisation were as follows: gas temperature 350 °C, drying gas flow rate of 11 l min^{-1}, nebuliser pressure 35 psi, and capillary voltage 4 kV. All ions were scanned in positive ion mode and given a dwell time of 50 msec.

The fragmentor voltage and collision energies had previously been optimised for each compound and can be found in Supplementary Table S2. Data analysis was undertaken using Agilent Mass Hunter Quantitative analysis software for QQQ (Version B.07.01). Accurate quantification used the stable isotope labelled internal standards added during sample extraction, and data was normalised to the dry weight of the samples.

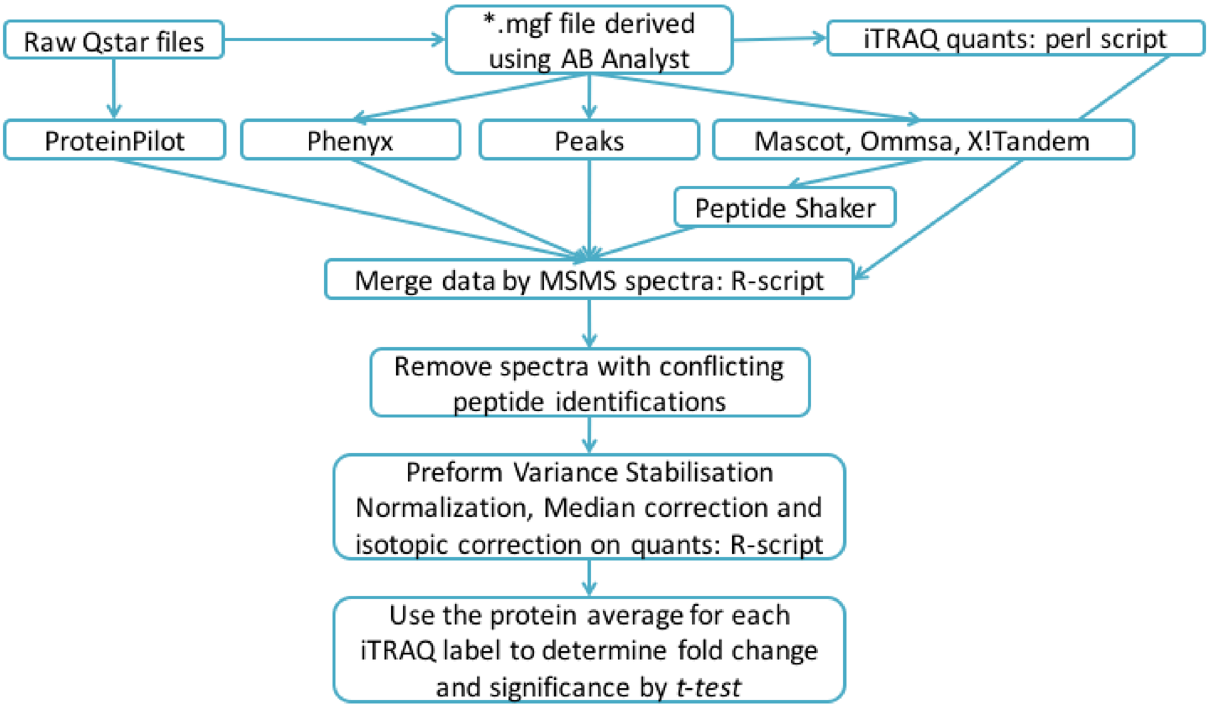

Fig. S1 Proteomics work flow

Rationale of the iTRAQ workflow and analysis, as described in Longworth et al. 2016. Proteome response of *Phaeodactylum tricornutum*, during lipid accumulation induced by nitrogen depletion. *Algal Research* **18:** 213-224

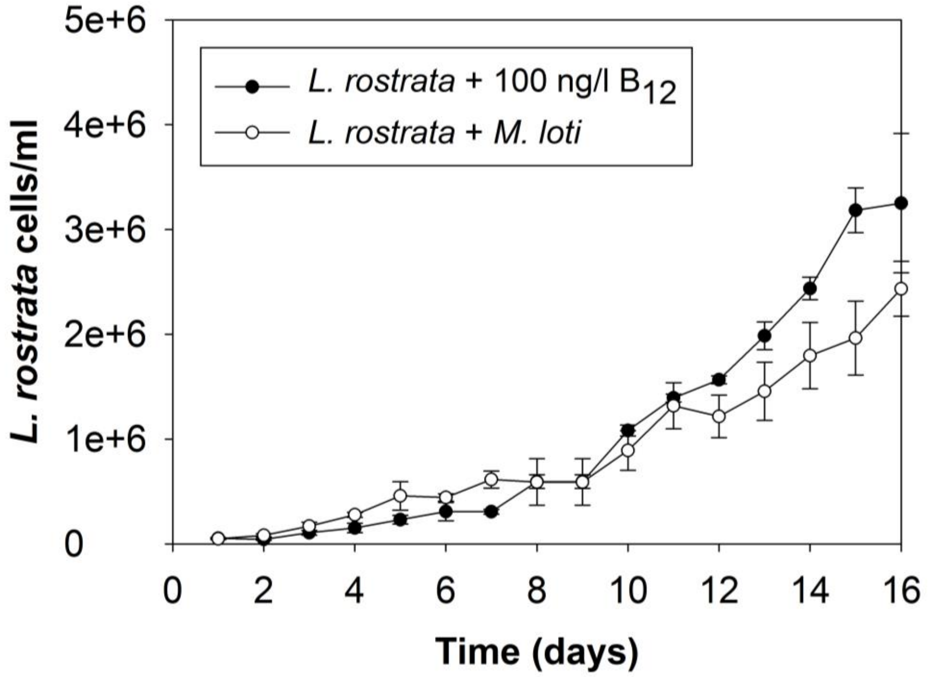

Fig. S2. Growth over time of *L. rostrata* grown in axenic culture with B_{12} supplementation (100 ng/l) versus in co-culture with *M. loti.* Data are the mean of 3 replicates ± SEM

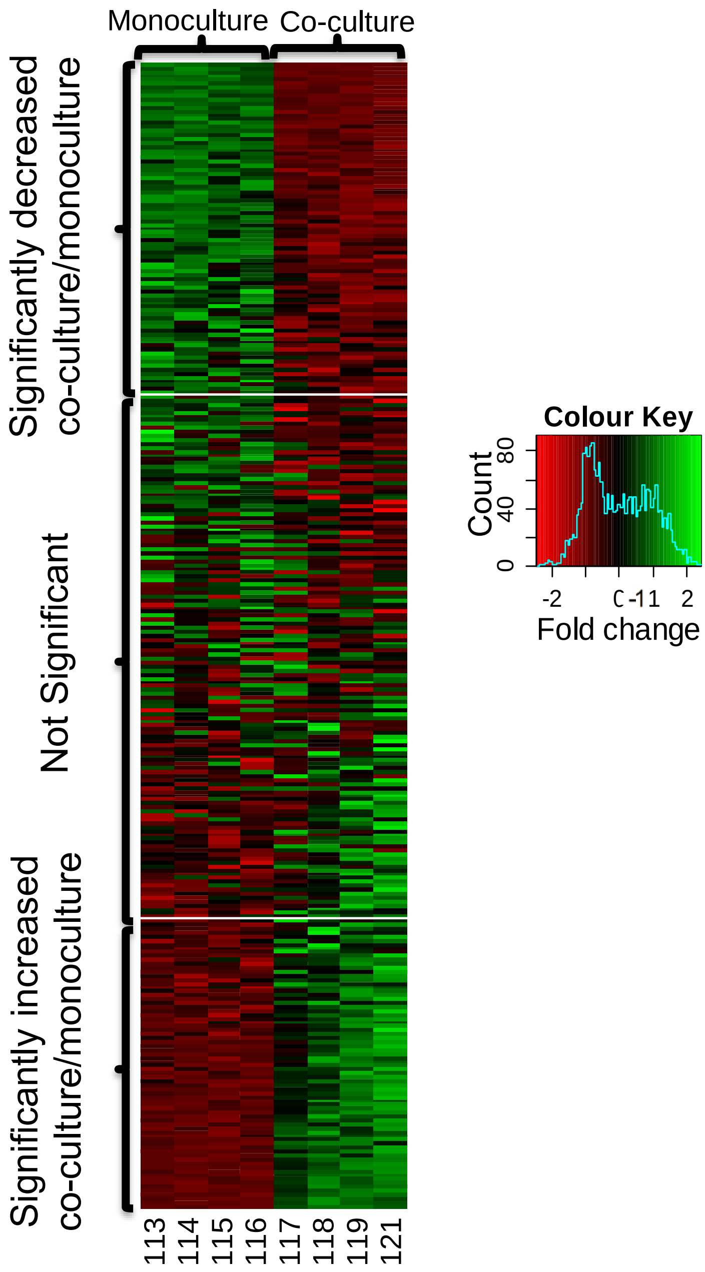

Fig. S3 Heatmap of proteins identified from iTRAQ analysis : Shown is the observed protein abundance for the 293 proteins that were detected with >2 unique peptides, ordered by direction and significance of change observed. A total of 83 proteins showed reduced abundance in co-cultures versus monocultures with B_{12} (p-value < 0.05), and 70 showed increased abundance. The clustering shows good consistency between biological replicates. Key: 113, 114, 115 and 116 are for *L. rostrata* cells grown axenically with 100 ng/L B_{12} (monoculture); 117, 118, 119 and 121 are for cells of cocultures of *L. rostrata* cells with *M. loti*.

Table S1 List of primers used for reverse transcriptase-quantitative polymerase chain reaction (RT-qPCR). Sequences were derived from RNAseq data from *Lobomonas rostrata* (unpublished) annotated by sequence similarity to homologues from *Chlamydomonas reinhardtii.*

Gene encoding	Code	Primer name	Sequence
Ribose-5-phosphate isomerase	RPI1	Lros_RPI1_F	CTGCTGCCGGATGAGACGTC
Ribose-5-phosphate isomerase	RPI1	Lros_RPI1_R	CCCTCAGCCAGCACCACAAA
Sedoheptulose-1,7-bisphosphatase	SEBP1	Lros_SEBP1_F	TGTGCAAGTACGCCTGCTCC
Sedoheptulose-1,7-bisphosphatase	SEBP1	Lros_SEBP1_R	GGTGATGCCGGTGAGCTTGT
Fructose-1,6-bisphosphate aldolase	FBA1	Lros_FBA1_F	AACCAGAAGCCCAACCCGTG
Fructose-1,6-bisphosphate aldolase	FBA1	Lros_FBA1_R	GGGTCGTACTTGCCCTGCTG
Ribulose phosphate-3-epimerase	RPE1	Lros_RPE1_F	GTCGTCCACCATCCACCTGC
Ribulose phosphate-3-epimerase	RPE1	Lros_RPE1_R	CCGGGGTTGACGCTCATGAT
Glyceraldehyde-3-phosphate dehydrogenase	GAP1a	Lros_GAP1A_F	CGACCTGACGCTCAACCTGG
Glyceraldehyde-3-phosphate dehydrogenase	GAP1a	Lros_GAP1A_R	CATGATGCCGGCCTTGGAGT
Glyceraldehyde-3-phosphate dehydrogenase	GAP3	Lros_GAP3_F	ATGCCGTTGAGCTTGCCCTT
Glyceraldehyde-3-phosphate dehydrogenase	GAP3	Lros_GAP3_R	ACCCACTCCTACACTGGCGA
Phosphoglycerate kinase	PGK1	Lros_PGK1_F	AGGTGAAGATCATGCCGCCG
Phosphoglycerate kinase	PGK1	Lros_PGK1_R	ACCAAGTTCCTCAAGCCCGC
Fructose-1,6-bisphosphatase	FBP1	Lros_FBP1_F	CTTGCCACCCCACTTCTCGG
Fructose-1,6-bisphosphatase	FBP1	Lros_FBP1_R	TGGTCGGCGAGTTTGTGCTC
Autophagy-related protein	APG8	Lros_APG8_F	CGTCCTCATCTCGGTGGTCC
Autophagy-related protein	APG8	Lros_APG8_R	GAAGAGCGACATCCCCGACA
Carbonic anhydrase	CAH1	Lros_CAH1_F	TGGTGTTGGTGGTGGCGC
Carbonic anhydrase	CAH1	Lros_CAH1_R	TACCTACGCTGGCTCGCTCA
Hydroxymethylpyrimidine phosphate synthase	THICb	Lros_THICb_F	CGTGATGTCGTACTTGGCGC
Hydroxymethylpyrimidine phosphate synthase	THICb	Lros_THICb_R	CAGGGCGTCGACTACTGGAC
Receptor of activated protein kinase C	RACK1	Lros_RACK1_F	CAACACCGTCACCGTCTCCC
Receptor of activated protein kinase C	RACK1	Lros_RACK1_R	GCGGTTGGGCGAGAAGATGA
Eukaryotic translation initiation factor 4A	EIF4A	Lros_EIF4A_F	ACAGCGTGTCCACTTCCAC
Eukaryotic translation initiation factor 4A	EIF4A	Lros_EIF4A_R	GCTGCAGGTCGGTGTGTTCT
Ubiquitin	UBQ	Lros_UBQ_F	CCTCACGGGCAAGACTATCA
Ubiquitin	UBQ	Lros_UBQ_R	GATGTTGTAGTCAGCCAGCG

Table S2 Optimised values for Mass-spectroscopic analysis of amino acids

MRM - Positive Polarity

Compound Name	Precursor Ion	Product Ion	Dwell	Frag-mentor	Collision Energy	Cell Accel-erator Voltage
Alanine	260.1	171	50	27	21	4
Alanine (L)	264.1	171	50	27	21	4
Arginine	345.1	171	50	27	17	4
Arginine (L)	355.1	171	50	27	17	4
Asparagine	303.1	171	50	24	21	4
Asparagine (L)	309.1	171	50	24	21	4
Aspartic Acid	304.1	171	50	27	23	4
Aspartic Acid (L)	309.1	171	50	27	23	4
Cysteine	292	171	50	27	21	4
Cysteine (L)	296	171	50	27	21	4
Cystine (1tag)	411	171	50	20	18	4
Cystine (2tags)	581.64	171	50	20	18	4
Glutamic Acid	318.1	171	50	27	21	4
Glutamic Acid (L)	324.1	171	50	27	21	4
Glutamine	317.1	171	50	22	24	4
Glutamine (L)	324.1	171	50	22	24	4
Glycine	246.1	171	50	27	21	4
Glycine (L)	249.1	171	50	27	21	4
GSH	478.1	171	50	30	20	4
GSSG	783.2	171	50	30	20	4
GSSG (2)	953.2	171	50	30	20	4
Histidine	326.1	171	50	18	12	4
Histidine (L)	335.1	171	50	18	12	4
Homoserine	290.1	171	50	30	20	4
Isoleucine	302.1	171	50	28	21	4
Isoleucine (L)	309.1	171	50	28	21	4
Leucine	302.1	171	50	27	20	4
Leucine (L)	309.1	171	50	27	20	4
Lysine	487.21	171	50	18	18	4
Lysine (L)	495.58	171	50	18	18	4
Methionine	320.1	171	50	27	21	4
Methionine (L)	326.1	171	50	27	21	4
Phenylalanine	336.1	171	50	29	21	4
Phenylalanine (L)	346.1	171	50	29	21	4
Proline	286.1	171	50	23	21	4
Proline (L)	292.1	171	50	23	21	4
Pyroglutamate	300	171	50	30	20	4
Serine	276.1	171	50	25	19	4
Serine (L)	280.1	171	50	25	19	4

Taurine	296	171	50	18	15	4
Threonine	290.1	171	50	25	20	4
Threonine (L)	295.1	171	50	25	20	4
Tryptamine	331.1	171	50	30	20	4
Tryptophan	375.1	171	50	30	25	4
Tryptophan (L)	388.1	171	50	30	25	4
Tyrosine	352.1	171	50	30	20	4
Tyrosine (L)	362.1	171	50	30	20	4
Valine	288.2	171	50	28	21	4
Valine (L)	294.1	171	50	28	21	4
